# Icariin inhibits hypoxia/reoxygenation‐induced ferroptosis of cardiomyocytes via regulation of the Nrf2/HO‐1 signaling pathway

**DOI:** 10.1002/2211-5463.13276

**Published:** 2021-09-27

**Authors:** Xiu‐Juan Liu, Yan‐Fei Lv, Wen‐Zhu Cui, Yan Li, Yang Liu, Yi‐Tao Xue, Feng Dong

**Affiliations:** ^1^ Department of cardiovascular diseases Shandong University of Traditional Chinese Medicine Affiliated Hospital Jinan China; ^2^ Department of Rehabilitation Medicine Shandong Provincial Hospital affiliated to Shandong First Medical University Jinan China; ^3^ Department of cardiovascular diseases The Second Affiliated Hospital of Shandong University of Traditional Chinese Medicine Jinan China

**Keywords:** cardiomyocytes, ferroptosis, hypoxia/reoxygenation, icariin, Nrf2/HO‐1 pathway, oxidative stress

## Abstract

Myocardial infarction (MI) is caused by the formation of plaques in the arterial walls, leading to a decrease of blood flow to the heart and myocardium injury as a result of hypoxia. Ferroptosis is a crucial event in myocardial injury, and icariin (ICA) exerts protective effects against myocardial injury. Here, we investigated the protective mechanism of ICA in hypoxia/reoxygenation (H/R)‐induced ferroptosis of cardiomyocytes. H9C2 cells were subjected to H/R induction. The content of lactate dehydrogenase and the levels of oxidative stress and intracellular ferrous ion Fe^2+^ were measured. The levels of ferroptosis markers (ACSL4 and GPX4) were detected. H/R‐induced H9C2 cells were cultured with ICA in the presence or absence of ferroptosis inducer (erastin). Znpp (an HO‐1 inhibitor) was added to ICA‐treated H/R cells to verify the role of the Nrf2/HO‐1 pathway. H/R‐induced H9C2 cells showed reduced viability, enhanced oxidative stress and lactate dehydrogenase content, increased levels of Fe^2+^ and ACSL4, and decreased levels of GPX4. ICA inhibited H/R‐induced ferroptosis and oxidative stress in cardiomyocytes. Erastin treatment reversed the inhibitory effect of ICA on ferroptosis in H/R cells. The expression of Nrf2 and HO‐1 in H/R‐induced H9C2 cells was reduced, whereas ICA treatment reversed this trend. Inhibition of the Nrf2/HO‐1 pathway reversed the protective effect of ICA on H/R‐induced ferroptosis. Collectively, our results suggest that ICA attenuates H/R‐induced ferroptosis of cardiomyocytes by activating the Nrf2/HO‐1 signaling pathway.

## Graphical abstract

Hypoxia/reoxygenation can induce ferroptosis in cardiomyocytes. Here, we report that icariin can activate the Nrf2/HO‐1 signaling pathway and inhibit hypoxia/reoxygenation‐induced ferroptosis in cardiomyocytes.

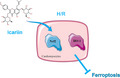

**Abbreviations**
CAT: catalase
CCK‐8: Cell Counting Kit‐8
DCFH‐DA: 2,7‐dichlorodihydrofluorescein diacetate
DMEM: Dulbecco's modified Eagle's medium
H/R: hypoxia/reoxygenation
ICA: icariin
LDH: lactate dehydrogenase
MDA: malondialdehyde
MI: myocardial infarction
MI/R: myocardial ischemia–reperfusion
ROS: reactive oxygen species

Myocardial infarction (MI) is a cardiac event caused by the formation of plaques in the arterial walls leading to the decrease of blood flow to the heart and injury of the myocardium as a result of hypoxia [1]. Currently, the most potent intervention strategy of MI is timely myocardial reperfusion, including thrombolytic therapy and primary percutaneous coronary intervention [2]. These intervention measures can swiftly restore the blood circulation of ischemic myocardium, limit the size of MI and, importantly, avert the occurrence of myocardial failure [3, 4]. Nevertheless, the restoration of blood flow may result in additional cardiac injury and complication, especially the death of cardiomyocytes, termed myocardial ischemia–reperfusion (MI/R) injury [5]. MI/R injury has been increasingly demonstrated to be concerned with nonapoptotic pathways, such as necroptosis [6], pyroptosis [7] and ferroptosis [8]. Accordingly, targeting the cardiomyocyte death concerning MI/R injury is accepted as a prospective therapeutic strategy.

Ferroptosis constitutes a type of regulated cell death and is identified as iron‐dependent cell death [9]. Ferroptosis varies from the other classical nonapoptotic cell death processes in that it is characterized by mitochondrial contraction and enhanced mitochondrial membrane density (morphological), lipid peroxidation (biochemical) and implication of a unique group of genes (genetic) [10, 11]. The pathological involvement of ferroptosis in I/R injury has been well documented in recent literature, and suppression of ferroptosis contributes to protecting cells from I/R injury [12]. For example, repression of ferroptosis in a diabetes mellitus MI/R model can attenuate endoplasmic reticulum stress and alleviate myocardial injury [8]. Ferrostatin‐1 and iron chelation are conducive to ameliorating heart failure resulting from acute and chronic I/R [13], which is consistent with the view that targeting ferroptosis acts as a potential strategy for the prevention of cardiomyopathy. Therefore, exploring the specific mechanism of cardiomyocyte death induced by ferroptosis is pivotal for the effective attenuation of MI/R injury.

Icariin (ICA), a flavonoid extracted from epimedii, has been demonstrated to exert potential protective effects on the cardiovascular system [14]. For example, ICA pretreatment can significantly suppress cardiomyocyte apoptosis by inhibiting endoplasmic reticulum stress [15]. ICA represses cardiomyocyte apoptosis, and such an effect is partially achieved by inhibiting the reactive oxygen species (ROS)‐dependent JNK/NF‐κB signaling [16]. Importantly, emerging evidence has unveiled that ICA attenuates infarct size induced by I/R in rats and consequently may become a potent agent for angiogenic therapy [14, 17]. ICA bears cardioprotective effects against MI/R injury, and its mechanism is related to the antioxidant and antiapoptotic function of ICA [18]. However, whether ICA can protect hypoxia/reoxygenation (H/R)‐induced cardiomyocytes by inhibiting ferroptosis remains unknown. This study investigated the protective mechanism of ICA in H/R‐induced ferroptosis of cardiomyocytes, which shall confer novel insights into the management of MI/R injury.

## Materials and methods

### Cell culture

The rat cardiomyocyte H9C2 (American Type Culture Collection) was cultured in Dulbecco's modified Eagle's medium (DMEM) containing 10% fetal bovine serum, 100 U·mL^−1^ penicillin and 100 μg·mL^−1^ streptomycin. The cells were cultured in a humidified incubator with 95% air and 5% CO_2_ at 37 °C.

### Establishment of H/R model and cell treatment

The H/R model was established by the previous literature [19]. Cells were incubated in glucose‐free DMEM and placed in an anaerobic incubator (95% N_2_ and 5% CO_2_) at 37 °C for 4 h. Subsequently, the cells were cultured in DMEM containing 4.5 mm glucose and placed in an incubator (95% air and 5% CO_2_) at 37 °C for 24 h. H9C2 cells were treated with different concentrations of ICA or PBS or 3 mm Znpp (Sigma‐Aldrich, Merck KGaA, Darmstadt, Germany) [20] or 5 μm erastin (Tocris, Minneapolis, MN, USA) [21] for 24 h and then subjected to H/R treatment.

### Cell Counting Kit‐8 assay

The toxic effect of ICA on cardiomyocytes was measured using Cell Counting Kit‐8 (CCK‐8; Dojindo, Mashiki‐machi, Japan). H9C2 cells were treated with 2.5, 5, 10 and 20 μm ICA (Sigma‐Aldrich) for 24 h, respectively. Then the cells under different treatment were seeded into the 96‐well plates (1 × 10^4^ cells per well). Each well was supplemented with 10 μL CCK‐8 solution and incubated for 2 h. The absorbance at 450 nm was detected by the microplate reader (Synergy HT; BioTek Instruments Inc., Winooski, VT, USA) and expressed as control percentage.

### Detection of lactate dehydrogenase

The release of lactate dehydrogenase (LDH) was detected using LDH cytotoxicity assay kit (Roche, Mannheim, Germany) to evaluate the degree of H9C2 cell injury. The absorbance value at 492 nm was measured by a spectrometer (Lab Tech, Boston, MA, USA).

### Determination of ROS, SOD, malondialdehyde, catalase and intracellular ferrous ion (Fe^2+^)

The ROS production, malondialdehyde (MDA) level, SOD and catalase (CAT) activity, and Fe^2+^ in the cells under different treatments were measured using the corresponding kits. The cells were incubated with 2,7‐dichlorodihydrofluorescein diacetate (DCFH‐DA) probe in the dark for 30 min in line with the instructions of the ROS detection kit (Beyotime, Shanghai, China). Then the fluorescence intensity was observed under the fluorescent microplate. The laser wavelength was 485 nm, and the emission wavelength was 525 nm; ROS level (%) = fluorescence value of intervention group/control group × 100%. CAT activity was detected using visible spectrophotometry and CAT detection kit (A007‐1‐1; Jiancheng Bioengineering Institute, Nanjing, China). MDA level was determined using the thiobarbituric acid method and MDA detection kit (A003‐1‐2; Jiancheng Bioengineering Institute). SOD activity was detected using the hydroxylamine method and total SOD detection kit (A001‐1‐2; Jiancheng Bioengineering Institute). The absorbance value at 593 nm was measured to calculate the iron ion level in line with the instruction of Fe^2+^ iron ion kit (MAK025; Sigma‐Aldrich).

### Reverse transcription quantitative PCR

Total RNA was extracted from cardiomyocytes using TRIzol reagent (Invitrogen, Carlsbad, CA, USA). RNA concentration was measured using a spectrophotometer (NanoVue™; General Electric Company, Schenectady, NY, USA). The extracted RNA was reverse transcribed into cDNA using the kit (Invitrogen). Real‐time PCR was performed on ABI 7500 platform (Applied Biosystems, Carlsbad, CA, USA; Thermo Fisher Scientific, Waltham, MA, USA) using SYBR Green Mix (Invitrogen). The relative expression of gene was calculated based on the 2^−ΔΔCt^ method, with GAPDH as an internal reference [22]. Each experiment was repeated three times independently. Primer sequences were shown in Table 1.

**Table 1:** Primer sequences for reverse transcription quantitative PCR. F, forward; R, reverse.

Name of primer	Sequences (5′–3′)
Nrf2‐F	ATGATGGACTTGGAATTGCCACCG
Nrf2‐R	CTAGTTTTTCTTTGTATCTGGC
HO‐1‐F	ATGGAGCGCCCACAGCTCGACA
HO‐1‐R	TTACATGGCATAAATTCCCACTGC
GAPDH‐F	ATGGTGAAGGTCGGTGTGAACGGA
GAPDH‐R	TTACTCCTTGGAGGCCATGTAGGC

### Western blot analysis

Total protein was extracted from H9C2 cells using the total protein extraction kit (Applygen Technologies, Beijing, China), and protein concentration was examined using bicinchoninic acid kit (Beyotime). An equal amount of protein (30 μg) was separated by 12% SDS/PAGE and then transferred onto polyvinylidene fluoride membranes (Millipore, Billerica, MA, USA). The membranes were blocked with 5% skim milk for 1 h and incubated with the primary antibodies GPX4 (1 : 1000, ab125066; Abcam, Cambridge, MA, USA), ACSL4 (1 : 10 000, ab155282; Abcam), Nrf2 (1 : 1000, ab92946; Abcam), HO‐1 (1 : 2000, ab189491; Abcam) and β‐actin (1 : 1000, ab8227; Abcam) at 4 °C overnight. Afterward, the membranes were incubated with horseradish peroxidase‐labeled secondary antibody (1 : 2000, ab205718; Abcam) for 1 h. The protein band was visualized using enhanced chemiluminescence system (Thermo Fisher Scientific). The band intensity was analyzed using imagej software (NIH Image, Bethesda, MD, USA).

### Statistical analysis

Data were analyzed and introduced using spss 21.0 (IBM Corp., Armonk, NY, USA) and graphpad prism 8.0 (GraphPad Software, San Diego, CA, USA). Data are expressed as mean ± standard deviation. The Shapiro–Wilk test was used to test normal distribution. The *t* test was adopted for comparison between two groups. One‐way ANOVA or two‐way ANOVA was used for the comparisons among multiple groups, following Tukey's multiple comparisons test. A *P* value <0.05 represented statistical significance.

## Results

### H/R induced ferroptosis of cardiomyocytes

Ferroptosis is an iron‐dependent necrosis caused by iron overload [9, 23]. Ferroptosis can occur in I/R injury [24]. The effect of H/R on ferroptosis has been reported [25, 26, 27], but the mechanism is still elusive.

**Fig. 1:**
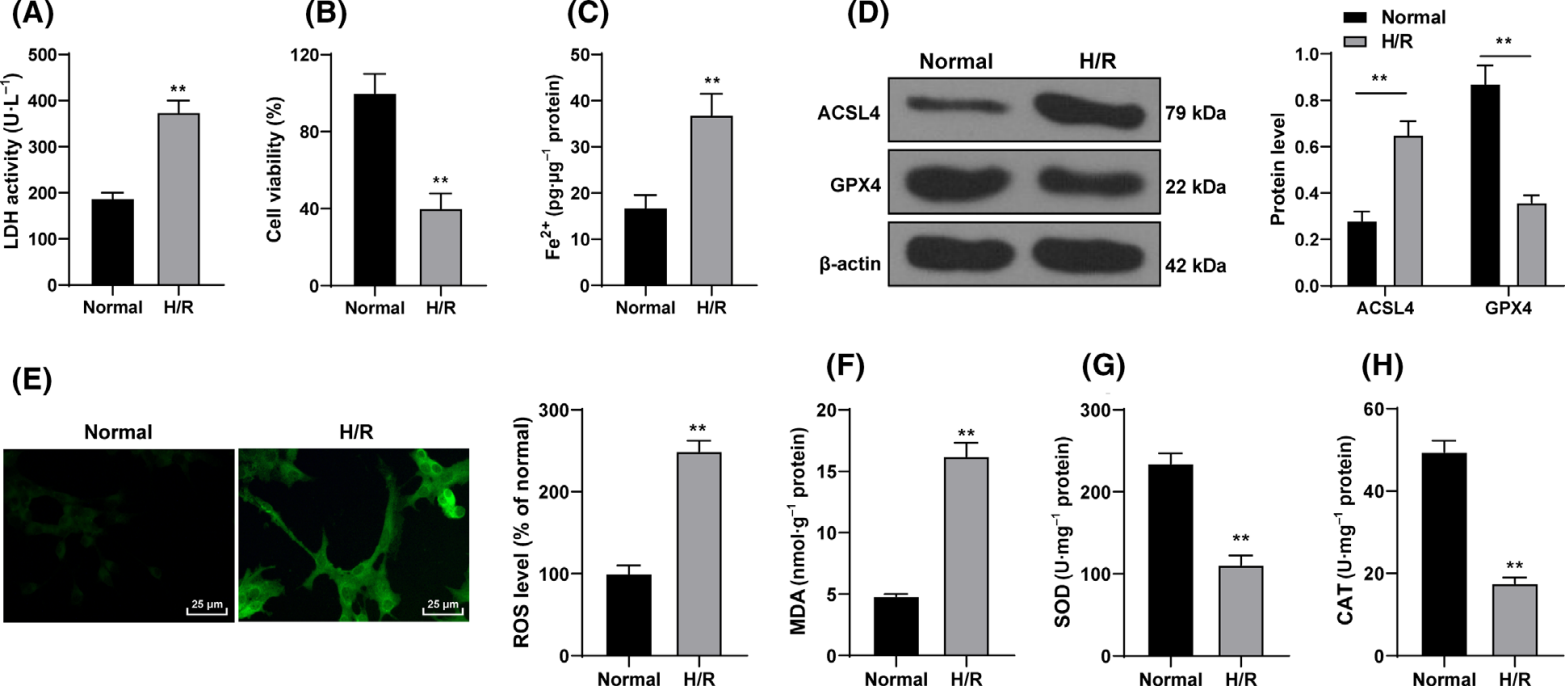
H/R‐induced ferroptosis of cardiomyocytes. H9C2 cells were subjected to H/R stimulation. (A) LDH content was detected using the LDH cytotoxicity assay kit. (B) Cell viability was examined using the CCK‐8 assay. (C) Fe^2+^ content was detected using the kit. (D) Ferroptosis‐related proteins GPX4 and ACSL4 were detected using western blot. (E) Fluorescence intensity of ROS was detected using DCFH‐DA probe. Scale bars, 25 μm. (F) The level of MDA in cells was detected by the thiobarbituric acid method and MDA assay kit. (G) Detection of SOD activity by the hydroxylamine method and total SOD assay kit. (H) The level of CAT was detected using the visible light method and CAT test kit. The cell experiment was repeated three times. Data were presented as mean ± standard deviation. Data in (A)–(C) and (E)–(H) were analyzed using *t* test, and data in (D) were analyzed using two‐way ANOVA, followed by Tukey's multiple comparison test, **P < 0.01.

In this study, H9C2 cells were subjected to H/R induction. After H/R, the cells showed increased LDH content (*P* < 0.01; Fig. 1A), decreased cell viability (*P* < 0.01; Fig. 1B) and increased Fe^2+^ content (*P* < 0.01; Fig. 1C). GPX4 and ACSL4 were used as markers of ferroptosis [21]. After H/R induction, ACSL4 expression was increased and GPX4 expression was decreased (*P* < 0.01; Fig. 1D). SOD, MDA and CAT are oxidative stress‐related factors [8, 28]. The results demonstrated that the fluorescence level of ROS in cells was significantly increased (*P* < 0.01; Fig. 1E), levels of MDA were increased, and the activities of SOD and CAT were decreased after H/R treatment (*P* < 0.01; Fig. 1F–H). Taken together, H/R treatment induced ferroptosis of cardiomyocytes.

### ICA inhibited H/R‐induced ferroptosis of cardiomyocytes

**Fig. 2:**
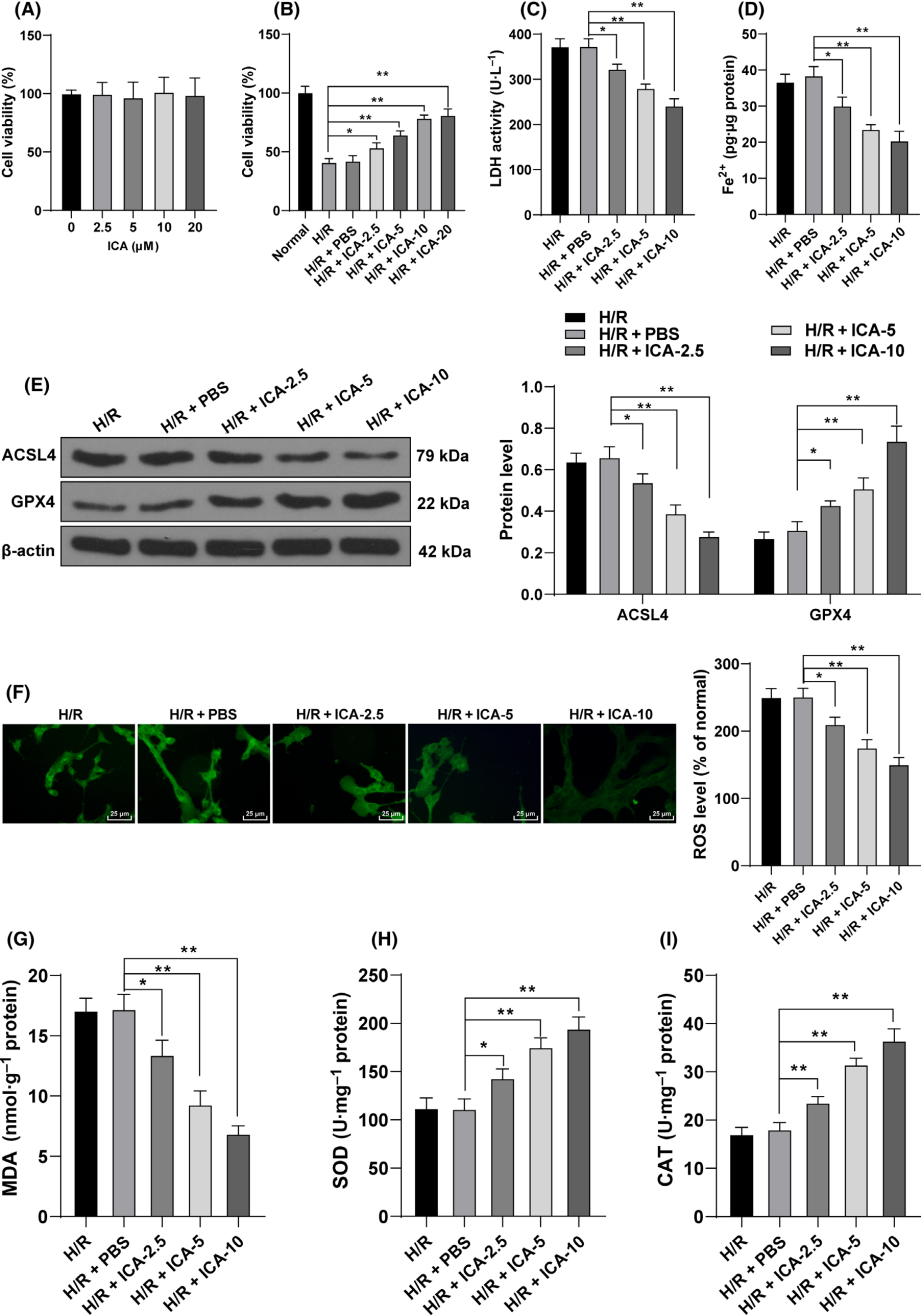
ICA inhibited H/R‐induced ferroptosis of cardiomyocytes. H/R‐induced cardiomyocytes were treated with ICA. (A, B) Cell viability was measured using CCK‐8 assay. (C) LDH content was detected. (D) Fe^2+^ content was detected. (E) Ferroptosis‐related proteins GPX4 and ACSL4 were detected using western blot. (F) Fluorescence intensity of ROS was detected using DCFH‐DA probe. Scale bars, 25 μm. (G–I) The level of MDA and the activities of SOD and CAT were detected. The cell experiment was repeated three times. Data were presented as mean ± standard deviation. Data in (A)–(D) and (F)–(I) were analyzed using one‐way ANOVA, and data in (E) were analyzed using two‐way ANOVA, followed by Tukey's multiple comparison test, *P < 0.05, **P < 0.01.

ICA exerts a protective effect on cardiomyocyte injury [29, 30, 31]. However, whether ICA has a protective effect on H/R‐induced ferroptosis remains unclear. The effect of ICA on cardiomyocyte viability was detected using CCK‐8 assay. The results exhibited that 2.5–20 μm ICA treatment did not affect H9C2 cell viability (*P* < 0.01; Fig. 2A), but the protective effect of ICA on H9C2 cells induced by H/R was dose dependent (2.5–10 μm), and there was no significant difference between the 10 μm and 20 μm groups (Fig. 2B). The ferroptosis of H/R cells treated with 2.5–10 μm ICA was detected. The results revealed that the contents of LDH and Fe^2+^ and the expression of ACSL4 were decreased with the increase of ICA concentration, while the expression of GPX4 was increased with the increase of ICA concentration (*P* < 0.05; Fig. 2C–E). In addition, compared with those in the H/R + PBS group, the ROS fluorescence intensity and MDA levels in the H/R + ICA group were decreased (*P* < 0.05; Fig. 2F,G), while the activities of SOD and CAT were increased, which were correlated with the concentration of ICA (*P* < 0.05; Fig. 2H,I). In brief, ICA inhibited H/R‐induced ferroptosis of cardiomyocytes, and the effect of ICA was enhanced with the increase of ICA concentration.

### ICA exerted a protective effect on H/R‐induced cardiomyocytes by inhibiting ferroptosis

**Fig. 3:**
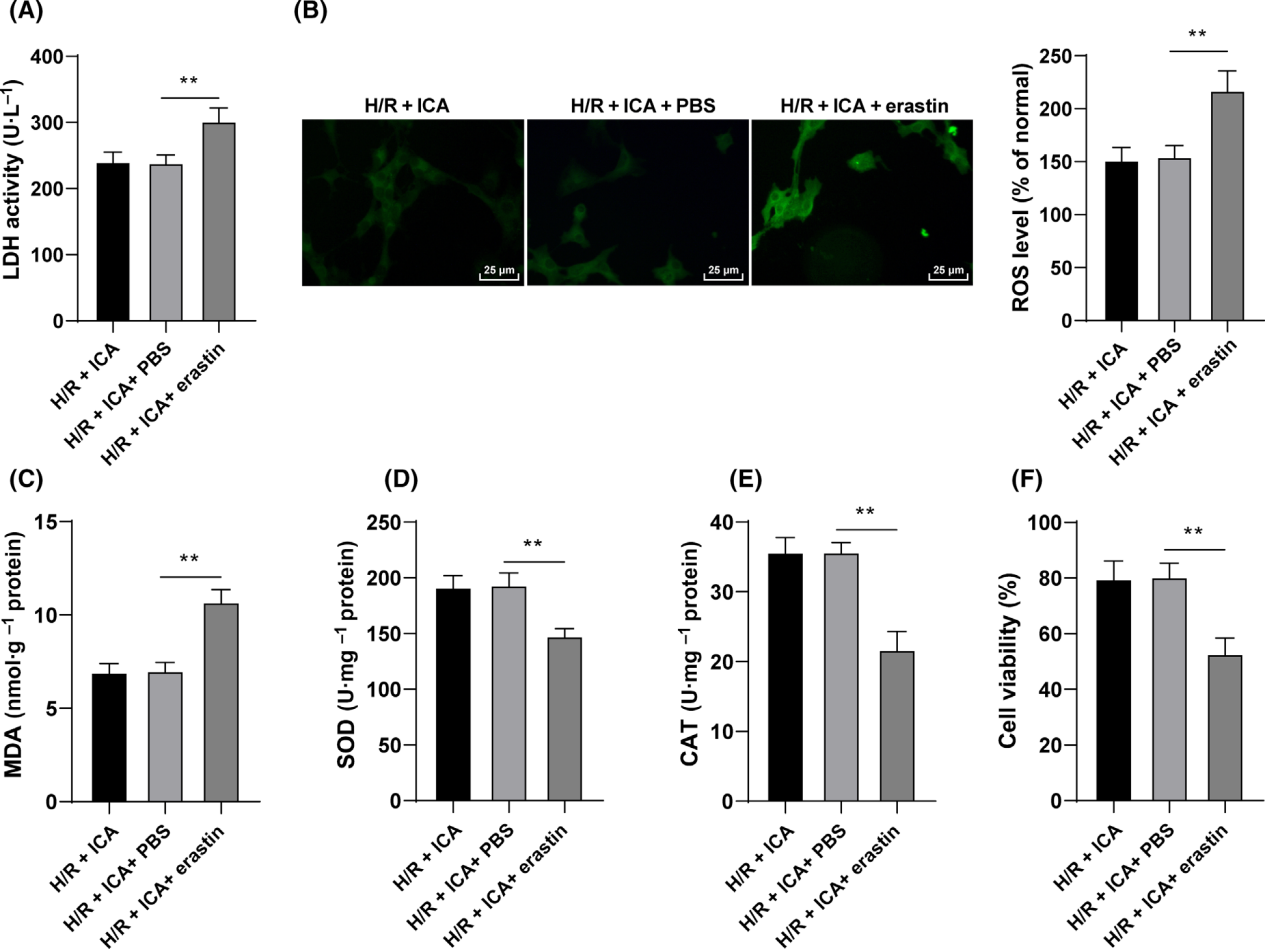
ICA exerted a protective effect on H/R‐induced cardiomyocytes by inhibiting ferroptosis. H/R‐induced cardiomyocytes were treated with 10 μm ICA and 5 μm erastin. (A) LDH content was detected. (B) Fluorescence intensity of ROS was detected using the DCFH‐DA probe. Scale bars, 25 μm. (C–E) The level of MDA and the activities of SOD and CAT were detected. (F) Cell viability was measured using CCK‐8 assay. The cell experiment was repeated three times. Data were presented as mean ± standard deviation. Data in (A)–(F) were analyzed using one‐way ANOVA, followed by Tukey's multiple comparison test, **P < 0.01.

To prove that ICA plays a protective role in H/R cardiomyocytes by regulating ferroptosis, we treated H/R cells with ferroptosis inducer erastin and ICA. Compared with the H/R + ICA + PBS group, the H/R + ICA + erastin group showed increased LDH content (*P* < 0.01; Fig. 3A). Moreover, the addition of erastin increased the ROS fluorescence intensity and MDA levels (*P* < 0.01; Fig. 3B,C), decreased the activities of SOD and CAT (*P* < 0.01; Fig. 3D,E), and reduced the viability of H/R cells (*P* < 0.01; Fig. 3F). These results suggested that induction of ferroptosis weakened the protective effect of ICA on H/R cardiomyocytes, and ICA played a protective role in H/R cardiomyocytes by regulating ferroptosis.

### ICA activated the Nrf2/HO‐1 pathway in H/R‐induced cardiomyocytes

**Fig. 4:**
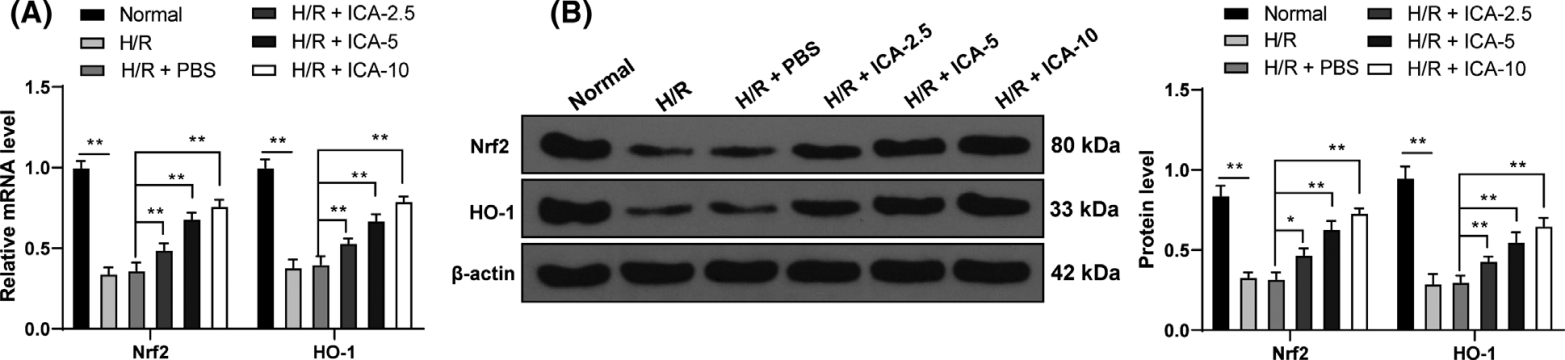
ICA activated the Nrf2/HO‐1 pathway in H/R‐induced cardiomyocytes. (A, B) The expressions of key factors of the Nrf2/HO‐1 signaling pathway, Nrf2 and HO‐1, were detected using reverse transcription quantitative PCR and western blot. The cell experiment was repeated three times. Data were presented as mean ± standard deviation and analyzed using two‐way ANOVA, followed by Tukey's multiple comparison test, *P < 0.05, **P < 0.01.

ICA can activate the Nrf2 pathway [32]. The Nrf2/HO‐1 signaling pathway is related to oxidative stress [33, 34]. Therefore, we speculated that ICA reduced ferroptosis of H/R cardiomyocytes by affecting the Nrf2/HO‐1 signaling pathway. The expressions of Nrf2 and HO‐1 were detected. The mRNA expressions and protein levels of Nrf2 and HO‐1 were reduced notably after H/R stimulation but were elevated with the increase of ICA concentration (*P* < 0.05; Fig. 4A,B). These results suggested that ICA treatment activated the Nrf2/HO‐1 pathway in H/R‐induced cardiomyocytes.

### Repression of the Nrf2/HO‐1 pathway attenuated the protective function of ICA on H/R‐induced ferroptosis

**Fig. 5:**
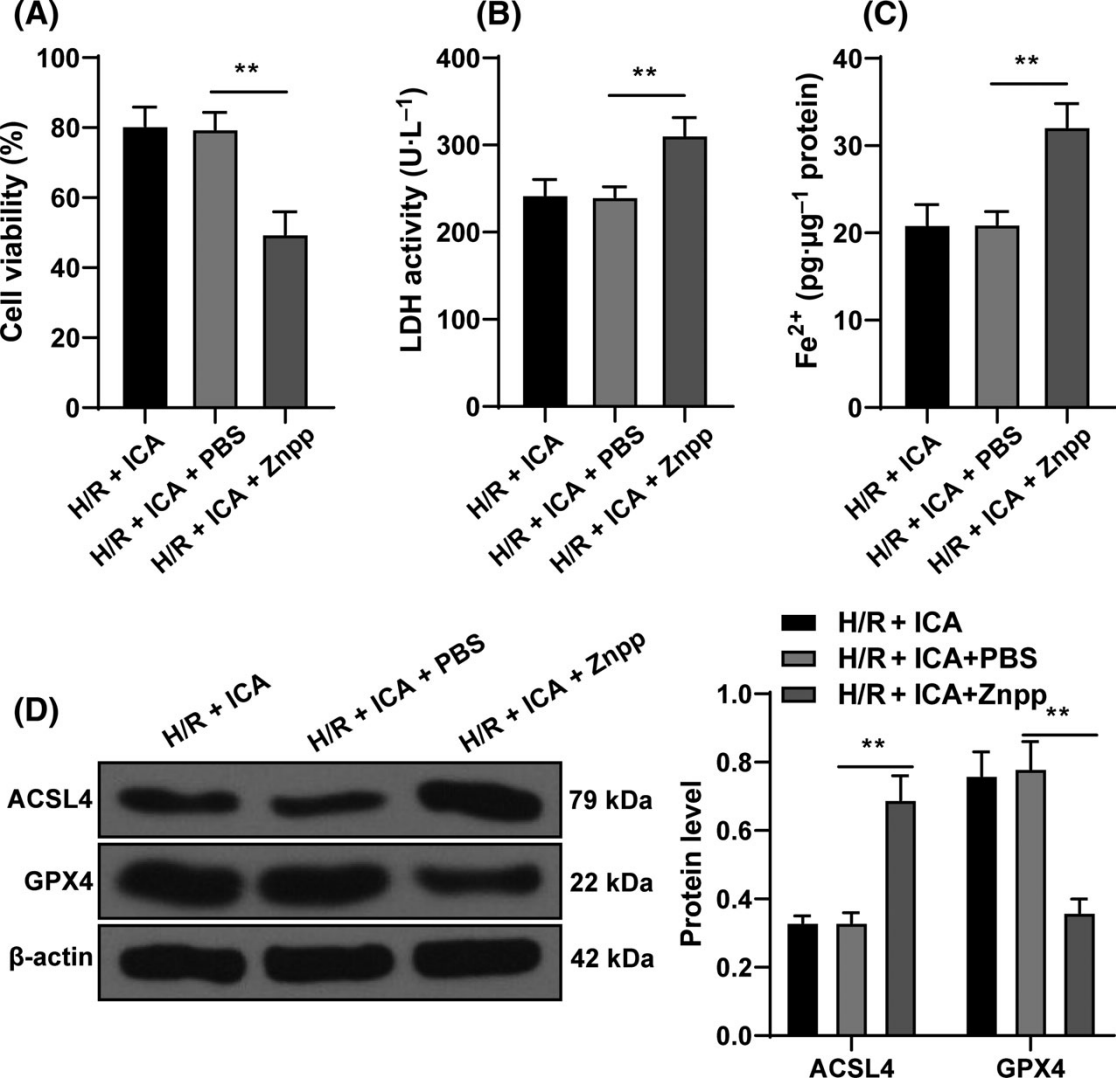
Inhibition of the Nrf2/HO‐1 pathway reduced the protective effect of ICA on H/R‐induced ferroptosis of cardiomyocytes. H/R‐induced cardiomyocytes were treated with 10 μm ICA and 3 mm Znpp. (A) Cell viability was measured using CCK‐8 assay. (B) LDH content in cells was detected. (C) Fe^2+^ content in cells was detected. (D) Ferroptosis‐related proteins GPX4 and ACSL4 were detected using western blot. The cell experiment was repeated three times. Data were presented as mean ± standard deviation. Data in (A)–(C) were analyzed using one‐way ANOVA, and data in (D) were analyzed using two‐way ANOVA, followed by Tukey's multiple comparison test, **P < 0.01.

To verify the role of the Nrf2/HO‐1 pathway, we conducted functional rescue experiments. Znpp (an HO‐1 inhibitor) was added to inactivate the Nrf2/HO‐1 pathway in ICA‐treated cells. Compared with the H/R + ICA + PBS group, the H/R + ICA + Znpp group had decreased cell viability, increased LDH content (*P* < 0.01; Fig. 5A,B), increased Fe^2+^ content and ACSL4 expression, and decreased GPX4 expression (*P* < 0.01; Fig. 5C,D). In brief, inhibition of the Nrf2/HO‐1 pathway reduced the protective effect of ICA on ferroptosis in H/R cardiomyocytes.

## Discussion

Myocardial I/R remains a serious complication of reperfusion therapy [35], which is related to a variety of pathophysiological characteristics, including cardiomyocyte ferroptosis [12]. ICA functions as a promising agent for alleviating MI/R injury [17]. This study demonstrated that ICA suppresses ferroptosis of H/R‐induced cardiomyocytes, thereby alleviating MI/R injury.

Because I/R injury is essentially concerned with oxidative damage, which is also one of the major causes of ferroptosis, accumulating studies have linked ferroptosis with I/R injury [12]. Targeting ferroptosis represents a promising strategy for the protection of I/R‐induced cardiomyopathy [13]. Still, the effect and mechanism of H/R on ferroptosis are unclear. In this study, H9C2 cells were subjected to H/R induction. During I/R, the integrity of myocardial membrane is lost, and myocardial enzymes including LDH are released into plasma; consequently, the enzyme level can be used as an indicator of myocardial injury [36]. Consistently, we showed that after H/R induction, the cells had increased LDH content, decreased cell viability and increased Fe^2+^ content. GPX4 is a crucial regulator of ferroptosis that protects cells by neutralizing lipid peroxides [37]. Direct repression of GPx4 or indirect repression by consumption of its substrate glutathione or glutathione components (such as cysteine) can induce ferroptosis [38]. ACSL4 is a critical enzyme that modulates lipid composition, contributing to the initiation of ferroptosis [39]. We showed that after H/R induction, ACSL4 expression in H9C2 cells was increased and GPX4 expression was decreased. Ferroptosis is identified to be associated with an oxidative stress‐induced cell death [40]. Excessive ROS results in oxidative stress and the subsequent generation of free radicals, which may damage DNA, proteins and lipids; ROS‐induced lipid peroxidation contributes to ferroptosis [23]. MDA is a product of lipid peroxidation and used as a marker of oxidative stress [41]. Oxidative stress is an imbalance between the generation of ROS and the activity of antioxidants, and the general endogenous antioxidant system is composed of enzymatic antioxidants, such as SOD and CAT [42]. The levels of ROS and MDA in H9C2 cells were increased, and the activities of SOD and CAT were decreased after H/R treatment. Taken together, H/R treatment induced oxidative stress and ferroptosis of cardiomyocytes.

ICA bears wide pharmacological activities, including anti‐inflammation and antioxidative stress [43], and importantly, it is identified to possess cardioprotective effects against MI/R injury [14, 30]. However, whether ICA shows a protective effect on H/R‐induced ferroptosis remains unclear. In this study, the protective function of ICA on H9C2 cells induced by H/R was dose dependent (2.5–10 μm). Hence we detected the ferroptosis of H/R cells treated with 2.5–10 μm ICA. The results revealed that the contents of LDH and Fe^2+^ and the expression of ACSL4 were decreased with the increase of ICA concentration, while the expression of GPX4 was increased with the increase of ICA concentration. In addition, compared with the H/R + PBS‐treated cells, the H/R + ICA‐treated cells had decreased levels of ROS and MDA and increased activities of SOD and CAT. In brief, ICA inhibited H/R‐induced ferroptosis of cardiomyocytes, and the effect of ICA was enhanced with the increase of ICA concentration. Then we treated H/R‐induced cells with ferroptosis inducer erastin and ICA. After the addition of erastin, the LDH content of H/R‐induced cells was increased, the levels of ROS and MDA were increased, the activities of SOD and CAT were suppressed, and the cell viability was notably reduced. These results suggested that ICA protects H/R‐induced cardiomyocytes by inhibiting ferroptosis. Consistently, ICA prevents lipopolysaccharide‐induced cell death in synoviocytes by suppressing ferroptosis [44].

Thereafter, we explored the signaling pathway of ICA inhibiting ferroptosis. Accumulating studies have indicated that ICA plays a role in the process of diseases by activating the Nrf2 signaling [32, 45, 46]. For example, ICA attenuates oxidative stress in human lung epithelial cells by activating the Nrf2 signaling [47]. Nrf2 represents the critical mediator of the endogenously induced defense system, which translocates to the nucleus and binds to specific DNA sites in response to oxidative stress, thereby initiating the transcription of cytoprotective genes such as *HO‐1* [32]. Nrf2 activation confers cardiac protection by up‐regulating antioxidant and anti‐inflammatory mechanisms [48]. Enhancing Nrf2 expression alleviates myocardial oxidative stress in a diabetic heart and attenuates MI/R injury [49]. Moreover, Nrf2 is a mitigator of lipid peroxidation and ferroptosis, and aberrant NRF2 signaling leads to the diseases concerned with increased lipid peroxidation and ferroptosis [50]. HO‐1 can degrade heme into carbon monoxide, biliverdin and ferrous iron, and provides cardioprotection via antiapoptotic and antioxidant effects [51]. HO‐1 is implicated in ferroptosis via its correlation with iron and antioxidant effects [52]. Therefore, we speculated that ICA reduced ferroptosis of H/R‐induced cardiomyocytes by affecting the Nrf2/HO‐1 pathway. The expressions of Nrf2 and HO‐1 were reduced notably after H/R stimulation but were increased with the elevation of ICA concentration. Functional rescue experiment confirmed that inhibition of Nrf2/HO‐1 signaling reduced the protective effect of ICA on ferroptosis of H/R‐induced cardiomyocytes. ICA prevents extracellular matrix generation and oxidative stress in experimental diabetic kidney disease via Nrf2 activation [53]. In brief, ICA treatment activated the Nrf2/HO‐1 signaling, thereby inhibiting ferroptosis of H/R‐induced cardiomyocytes.

In summary, ICA represses H/R‐induced cardiomyocyte oxidation and ferroptosis by activating the Nrf2/HO‐1 pathway. The main innovation of this study is the effect and mechanism of ICA on H/R‐induced ferroptosis in cardiomyocytes. This study verified the role of only Nrf2/HO‐1 signaling in ICA; whether there are other signaling pathways involved in ICA protection was unclear. In addition, whether ICA could affect other factors, such as miRNA or mRNA, needs further exploration. In the future, we will verify the specific mechanism of other signaling pathways and whether the function of ICA can be used as the entry point of H/R cardiomyocytes, to provide some theoretical support for the MI/R injury.

## Conflict of interest

The authors declare no conflict of interest.

## Author contributions

XJU, YFL and FD: paper design, experimental analysis, data collection and analysis, and final writing. YFL: Data collection and analysis. WZC, YL and YTX: Experimental analysis and data statistics. FD: Financial Support and statistical analysis.

## Data Availability

The raw data supporting the conclusions of this article will be made available by the authors upon request without undue reservation.
